# Exosome-based therapy for epilepsy: a systematic review and meta-analysis of preclinical studies

**DOI:** 10.3389/fnins.2026.1824183

**Published:** 2026-05-15

**Authors:** Yongna Yang, Yinping Wu, Jianghua Si, Guolong Zhang, Liping Dong, Heng Liu, Chengyang Su, Zhilong Yang

**Affiliations:** 1 Department of Neurology, Lanzhou First People’s Hospital, Second Clinical Medical College of Gansu University of Traditional Chinese Medicine, Lanzhou, Gansu, China; 2 Department of Health Management Center, Lanzhou First People’s Hospital, Second Clinical Medical College of Gansu University of Traditional Chinese Medicine, Lanzhou, Gansu, China; 3 Department of Neurosurgery, Lanzhou First People’s Hospital, Second Clinical Medical College of Gansu University of Traditional Chinese Medicine, Lanzhou, Gansu, China

**Keywords:** animal experiments, efficacy, epilepsy, exosomes, meta-analysis

## Abstract

**Objective:**

This study aims to quantitatively assess the efficacy of exosome therapy for epilepsy through a systematic review and meta-analysis of preclinical animal experiments. We seek to clarify its overall effects on seizure reduction, cognitive function preservation, and neuroinflammation suppression.

**Methods:**

A systematic search was conducted across four English-language and four Chinese databases to include epilepsy animal studies. Continuous outcomes were synthesized using standardized mean differences (SMD) and 95% confidence intervals (CI), with fixed or random effects models selected based on heterogeneity.

**Results:**

A total of eight preclinical studies were included. The overall meta-analysis revealed that exosome treatment significantly reduced the duration of seizures (SMD = −2.30, 95% CI −4.24 to −0.36), decreased the frequency of spontaneous recurrent seizures (SMD = −1.38, 95% CI −2.17 to −0.58), and prolonged the seizure latency (SMD = 1.49, 95% CI 0.08–2.90). In terms of cognitive function, exosomes significantly shortened the escape latency in the Morris water maze (SMD = −1.38, 95% CI −2.17 to −0.58), increased the percentage of time spent in the target quadrant (SMD = 3.69, 95% CI 0.30–7.08), and enhanced the number of platform crossings (SMD = 1.41, 95% CI 0.60–2.21), with no significant changes in swimming speed. Neuropathological analysis indicated that exosome treatment significantly increased the number of hippocampal neurons (SMD = 4.48, 95% CI 1.46–7.49) and markedly reduced levels of glial fibrillary acidic protein (GFAP) (SMD = −3.61, 95% CI −7.08 to −0.14), ionized calcium-binding adaptor molecule 1 (IBA-1) (SMD = −10.27, 95% CI −20.29 to −0.25), tumor necrosis factor-alpha (TNF-α) (SMD = −2.95, 95% CI −4.21 to −1.69), and interleukin-1 beta (IL-1β) (SMD = −7.39, 95% CI −14.64 to −0.13). Although some outcomes exhibited heterogeneity and publication bias, the corrected primary effects remained statistically significant. The source of exosomes, administration route, and dosage may be critical variables influencing their efficacy.

**Conclusion:**

Exosome therapy improves seizure phenotypes and protects cognitive function in epilepsy models by suppressing neuroinflammation to promote neuronal survival, providing evidence for further mechanistic and clinical translation studies.

## 1 Introduction

Epilepsy is a chronic central nervous system (CNS) disorder characterized by abnormal, excessive, and synchronous neuronal discharges in the brain. Its global prevalence is approximately 6.38 per 1,000 population, with more than 3.2 million new cases each year and an estimated 51.7 million people affected worldwide, imposing a substantial disease burden on patients, families, and society (Fiest et al., 2017; GBD Epilepsy Collaborators, 2025). Current pharmacotherapy primarily suppresses seizures by modulating ion channels or neurotransmitter systems, yet it has limited capacity to intervene in the core pathological mechanisms of epileptogenesis, such as neuroinflammation, synaptic remodeling, and neuronal loss (Asadi-Pooya et al., 2023; Marson et al., 2021; Menon and Helen Cross, 2025). The US Food and Drug Administration (FDA) has approved multiple antiepileptic drugs (AEDs), including sodium valproate, levetiracetam, and lamotrigine. Although these agents effectively control seizures in some patients, approximately 30% still progress to drug-resistant epilepsy and often require combination therapy with two to four AEDs (Park et al., 2019). In addition, AEDs are commonly associated with adverse effects such as cognitive impairment, hepatotoxicity, rash, and teratogenicity, and they cannot reverse established abnormalities in neural networks (Meng et al., 2025; Wahab and Iqbal, 2023). Therefore, there is an urgent need in epilepsy research to explore novel therapeutic strategies that combine anticonvulsant, neuroprotective, and disease-modifying effects.

In recent years, regenerative medicine strategies, particularly those involving mesenchymal stem cells (MSCs), have garnered considerable attention due to their immunomodulatory and neuroprotective potential. However, the clinical application of live cell transplantation faces safety and ethical challenges, including immune rejection, microvascular occlusion, and potential tumorigenicity, which hinder its translational progress (Hoang et al., 2025). Exosomes, as key paracrine factors, not only inherit the therapeutic activity of their parent cells but also, due to their nanoscale size (30–150 nm), low immunogenicity, and natural ability to cross the blood-brain barrier, represent a promising “cell-free” therapeutic strategy (Mehdizadeh et al., 2025). Existing evidence suggests that exosomes can remodel the damaged brain microenvironment by delivering bioactive molecules, demonstrating unique advantages in inhibiting glial cell activation and the neuroinflammatory cascade (Liu et al., 2023). Compared with AEDs, exosomes may not only suppress acute seizures but also exert disease-modifying effects by regulating neuroinflammation and promoting neuroregeneration, potential that conventional AEDs do not offer. However, preclinical studies directly comparing the efficacy of AEDs and exosomes remain limited.

Despite the increasing number of animal studies investigating exosome therapy for epilepsy, the current evidence remains fragmented. There is considerable heterogeneity among studies regarding animal models, sources of exosomes, administration protocols, and outcome assessments. Additionally, individual animal experiments often lack statistical power due to limited sample sizes, leading to inconclusive findings regarding the precise efficacy of exosomes and optimal intervention strategies (Kumar et al., 2025; Movahedpour et al., 2023; Shaimardanova et al., 2022). Currently, there is a lack of comprehensive quantitative synthesis of existing preclinical evidence, which hampers an overall assessment of the potential efficacy of this therapy and obstructs the optimization of key parameters for clinical translation. Therefore, this study aims to quantitatively synthesize the preclinical evidence for exosome therapy in epilepsy through a systematic review and meta-analysis. We will focus on evaluating the comprehensive effects of exosomes on seizure control, cognitive protection, and neuroinflammation suppression, while also delving into the sources of heterogeneity, with the goal of providing critical parameter guidance and theoretical support for the transition of exosome therapy from the laboratory to clinical trials.

## 2 Materials and methods

### 2.1 Study design and registration

The design, implementation, and reporting of this study strictly adhere to the Preferred Reporting Items for Systematic Reviews and Meta-Analyses (PRISMA) statement. To ensure a high level of transparency and reproducibility in the research process, this study protocol has been registered with the International Prospective Register of Systematic Reviews (PROSPERO).

### 2.2 Inclusion and exclusion criteria

This study established rigorous screening criteria based on the PICOS (Population, Intervention, Comparison, Outcome, Study design) framework. The subjects were limited to established animal models of epilepsy or status epilepticus (SE), encompassing various rodent models induced by chemical agents (such as pilocarpine and kainic acid), electrical kindling, traumatic brain injury, or genetic modification, without restrictions on strain or sex. The intervention was defined as exosome treatment, including but not limited to exosomes derived from mesenchymal stem cells, dendritic cells, or engineered exosomes. Key information such as the source of exosomes, dosage, and administration routes (e.g., intravenous, hippocampal, or ventricular injection) must be clearly reported. Control groups included blank controls, vehicle/solvent controls, sham surgery controls, or cell culture supernatant without exosomes. The study design included controlled experiments.

To comprehensively assess the therapeutic potential of exosomes, we extracted predefined outcome measures from three dimensions: anticonvulsant effects, cognitive function protection, and microenvironment regulation. First, regarding seizure phenotype, we extracted seizure latency and tonic-clonic onset time as key indicators for evaluating acute anticonvulsant thresholds, while quantifying seizure severity through seizure duration and average seizure scores. Specifically, we extracted the frequency of spontaneous recurrent seizures (SRSs) and their latency to evaluate the anticonvulsant effects of the intervention. Second, in terms of cognitive behavioral function, we focused on hippocampal-dependent spatial learning and memory abilities, extracting escape latency, percentage of time spent in the target quadrant, and platform crossings from the Morris water maze experiment. To exclude the confounding effects of motor dysfunction due to epilepsy on cognitive testing, swim speed was also included as a key control variable for extraction and analysis. Finally, for neuropathology and the inflammatory microenvironment, we extracted the number of neurons in the hippocampus and cortex to evaluate neuroprotection. Notably, “extraction of neuron number” here refers to quantifying surviving neuronal counts in hippocampal or cortical regions from histological images (e.g., Nissl staining, NeuN immunofluorescence) reported in the original studies, rather than performing actual neuronal isolation. Additionally, quantitative data on glial fibrillary acidic protein (GFAP) and ionized calcium-binding adaptor molecule 1 (IBA-1) were evaluated to assess the degree of glial cell proliferation and activation, combined with levels of pro-inflammatory cytokines interleukin-1 beta (IL-1β) and tumor necrosis factor-alpha (TNF-α) to comprehensively evaluate the regulatory capacity of exosomes on neuroinflammatory cascades.

We excluded studies that employed non-randomized controlled designs, reviews, meta-analyses, conference abstracts, case reports, and *in vitro* cell studies. Studies utilizing non-epileptic disease models were also excluded. Research that could not provide full texts or critical original data (including those that remained inaccessible after contacting the authors) was excluded. Additionally, studies where the intervention was not exosomes or lacked clear descriptions of key characteristics (source, dosage) were excluded.

### 2.3 Literature search strategy

We systematically searched four English-language databases (PubMed, Embase, Web of Science, and the Cochrane Library) and four Chinese databases (CNKI, Wanfang, VIP, and Sinomed), from inception to 1 December 2025. The search strategy employed a combination of subject headings (MeSH/Emtree) and free-text terms using Boolean logic, including keywords related to “epilepsy” and “exosomes, extracellular vesicles.” The search queries for each database were adjusted according to their specific indexing rules (detailed search strategies are provided in Supplementary Table 1). Furthermore, to ensure comprehensive coverage, we manually searched the reference lists of included studies and relevant reviews in the field.

### 2.4 Literature screening and data extraction

Literature screening and data extraction were conducted independently by two researchers. All retrieved articles were imported into EndNote software, and duplicate records were removed. Initially, titles and abstracts were reviewed for preliminary screening, excluding studies that clearly did not meet the inclusion criteria. Subsequently, the full texts of the remaining studies were obtained for further screening to determine the final included literature. Any discrepancies were resolved through discussion or consultation with a third senior researcher. The entire screening process will be presented using a PRISMA flow diagram. Data extraction utilized a pre-designed standardized form, which included: ➀ basic study information; ➁ characteristics of the animal models; ➂ details of the intervention and control; ➃ means, standard deviations, and sample sizes for all predefined outcome measures. For graphical data, WebPlotDigitizer software was used for extraction. When studies reported the same outcome at multiple time points, the data from the final time point post-intervention were selected for analysis.

### 2.5 Risk of bias assessment

The methodological quality of the included studies was independently rated by two assessors using the Systematic Review Centre for Laboratory animal Experimentation (SYRCLE) risk of bias assessment tool for animal studies. This tool comprises 10 core items addressing selection bias (sequence generation, baseline characteristics balance, allocation concealment), performance bias (random housing, assessor blinding, researcher blinding), detection bias (random selection of outcome assessment, assessor blinding), attrition bias, and reporting bias. Each item was classified as “low risk,” “high risk,” or “unclear” based on the reporting in the original articles. Discrepancies in scoring were resolved through discussion or consultation with a third senior researcher.

### 2.6 Statistical analysis

All analyses were performed using R software (version 4.4.2) and its meta and metafor packages. All outcome measures were treated as continuous variables; due to potential differences in measurement methods and scales, standardized mean differences (SMD) and their 95% confidence intervals (CI) were used as the effect size for synthesis. Heterogeneity among studies was assessed using Cochran’s Q test (with a significance level set at *P* < 0.10) and the I^2^ statistic. If I^2^ ≤ 50% and *P* ≥ 0.10, a fixed-effects model was used for synthesis; if I^2^ > 50% or *P* < 0.10, a random-effects model was employed, and potential sources of heterogeneity were explored. To investigate heterogeneity, the following subgroup analyses were planned: ➀ source of exosomes; ➁ administration route; ➂ dosage of exosomes. For the outcome measure with the most studies, a funnel plot will be generated, and Egger’s test will be used to assess publication bias. If significant publication bias is detected, a trim-and-fill method will be applied for correction.

## 3 Results

### 3.1 Literature search results

**FIGURE 1:**
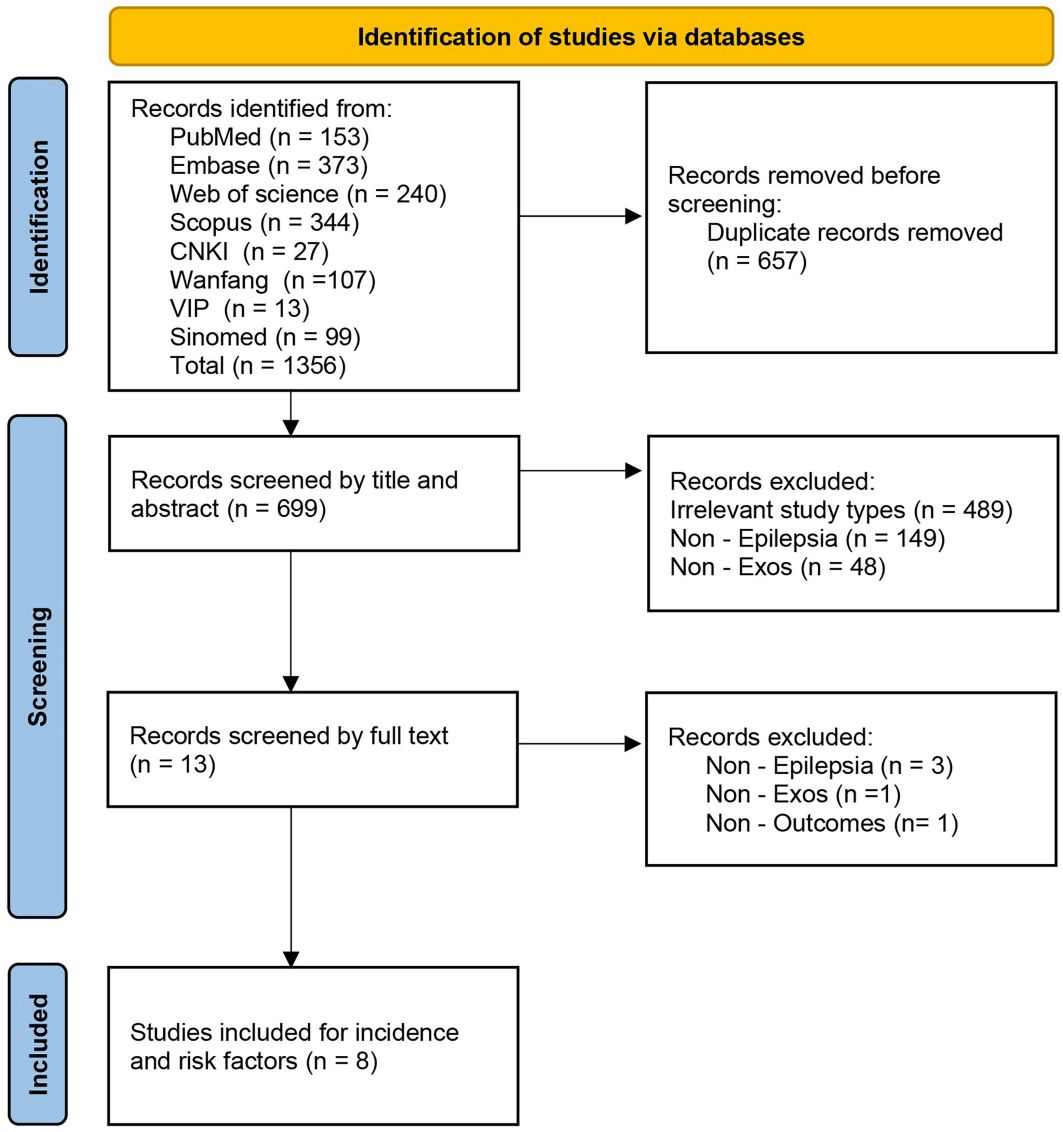
PRISMA 2020 flow diagram of study selection. Following a systematic search of eight Chinese and English databases, eight preclinical animal studies were ultimately included after deduplication, title/abstract screening, and full-text screening. The flow diagram details the numbers of records included and excluded at each stage and the reasons for exclusion.

A systematic search across eight Chinese and English databases initially yielded 1,356 relevant articles. After removing duplicates, 699 articles remained. A preliminary screening based on titles and abstracts excluded 489 studies unrelated to the research topic, 149 studies not related to epilepsy, and 48 studies not focused on exosomes, leaving 13 articles for full-text screening. Following a thorough review of the full texts, we further excluded 3 articles that were not related to epilepsy, 1 article that did not involve exosome interventions, and 1 article that lacked relevant outcome measures. Ultimately, a total of 8 preclinical animal studies meeting the inclusion criteria were included (Che et al., 2024; Derisfard et al., 2025; Ding et al., 2025; Liu et al., 2024; Long et al., 2017; Luo et al., 2021; Xian et al., 2019; Yang et al., 2024). The literature screening process adhered to the PRISMA 2020 guidelines, as illustrated in Figure 1.

### 3.2 Basic characteristics of included studies

This study included 8 randomized controlled animal experiments published between 2017 and 2025, conducted in three countries: China (6 studies), Iran (1 study), and the United States (1 study). All studies utilized mice to establish epilepsy models, with 6 specifically using the C57BL/6 strain. Seven studies employed male animals, while 1 study did not report the sex of the subjects. Induction methods included pilocarpine (four studies), kainic acid (three studies), and pentylenetetrazol (one study).

Based on seizure type and the assessment time window, the eight studies were categorized into three groups. Studies assessing only acute-phase seizure parameters (four studies) included those in which exosomes were administered via tail vein or intracerebroventricular injection after kainic acid (KA)- or pilocarpine-induced status epilepticus (SE) and evaluated in the acute phase, as well as an acute pentylenetetrazol (PTZ) seizure model with intranasal pretreatment followed by acute assessment. One study explicitly evaluated chronic spontaneous recurrent seizures (Ss): in an intrahippocampal KA-induced temporal lobe epilepsy model, exosomes were injected via the tail vein on days 1, 7, and 14 after KA, with continuous video-electroencephalography (video-EEG) monitoring performed on days 14–21. Studies assessing chronic pathological and cognitive outcomes without directly quantifying SRSs (three studies) administered exosomes intranasally, intracerebroventricularly, or via tail vein after pilocarpine-induced SE, and assessed cognition and histological changes in the chronic phase (5–8 weeks or 3 months) using the Morris water maze and electrophysiological evaluations. This categorization indicates that only a small proportion of studies (1/8) directly assessed the inhibitory effect of exosomes on chronic SRSs, whereas most focused on acute seizure control or chronic histopathology and cognitive protection. In addition, exosome sources were diverse. Human-derived exosomes accounted for six studies (75%), including exosomes from human umbilical cord mesenchymal stem cells (three studies; donors were healthy full-term parturients or healthy mothers aged 25–30 years; isolated by differential ultracentrifugation precipitation reagents), exosomes from human adipose-derived mesenchymal stem cells (two studies; donors were healthy female abdominal liposuction tissue; passages P4–P5; isolated using a polyethylene glycol (PEG) kit or density-gradient ultracentrifugation), and exosomes from human bone marrow mesenchymal stem cells (one study; bone marrow from healthy volunteers; isolated by chromatography and labeled as “A1-exosomes” after confirming anti-inflammatory activity in a lipopolysaccharide-stimulated splenic inflammation model). Mouse-derived exosomes accounted for two studies (25%), derived from mesenchymal stem cells from bone marrow/adipose tissue of C57BL/6J mice and from adipose-derived stem cells from adipose pads of normal C57BL/6 mice (passage P3), respectively. All studies performed basic exosome characterization (transmission electron microscopy, 8/8; size analysis, 6/8; marker protein detection, 7/8). Administration routes included tail vein injection (four studies), intranasal administration (two studies), and lateral ventricle/intracerebroventricular injection (two studies). Regarding dosing frequency, single-dose and multiple interventions each accounted for four studies; single-dose amounts ranged from 15 to 100 μg, and the total cumulative dose for multiple dosing ranged from 15 to 350 μg. For control treatment, all eight studies used saline or PBS. Sample sizes ranged from 4 to 12 animals per group, as detailed in Supplementary Table 2.

The sources of exosomes were predominantly adipose-derived (3 studies) and umbilical cord-derived (3 studies), with 2 studies utilizing bone marrow-derived exosomes. Administration routes included tail vein injection (4 studies), intranasal administration (2 studies), and lateral ventricle injection (2 studies). Among the dosing regimens, 5 studies involved single administration, while 3 studies employed multiple interventions, with total dosages ranging from 15 to 350 μg. All studies used saline or phosphate-buffered saline as control treatments, as detailed in Supplementary Table 2.

### 3.3 Risk of bias assessment results

**FIGURE 2:**
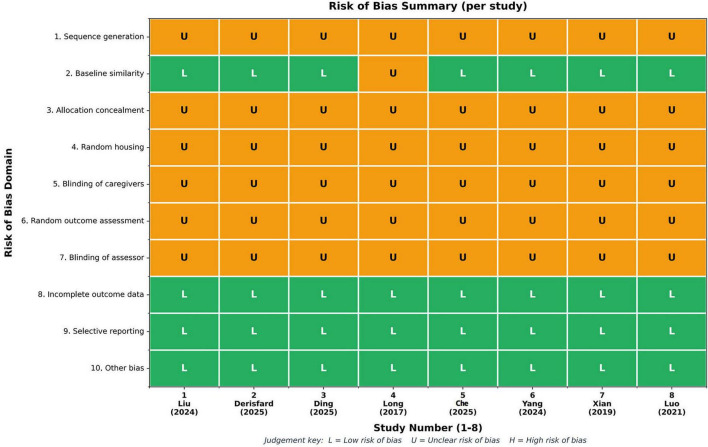
Summary of SYRCLE risk-of-bias assessments for included studies. The SYRCLE tool was used to evaluate the methodological quality of the eight included studies. The *x*-axis shows the eight included studies, and the *y*-axis shows the 10 risk-of-bias items. Green squares indicate “low risk,” yellow squares indicate “unclear risk,” and red squares indicate “high risk.” The figure provides an overview of the distribution of risks across selection bias, performance bias, detection bias, attrition bias, and reporting.

The risk of bias was unclear for the vast majority of studies. Specifically, regarding random sequence generation, allocation concealment, random housing, blinding of experimental implementation, random outcome assessment, and blinding of outcome evaluation, all 8 studies were assessed as having “unclear risk,” primarily due to insufficient descriptive information provided in the original publications. In terms of whether the groups were similar at baseline or if confounding factors were adjusted for in the analysis, 7 studies were rated as “low risk,” while 1 study was rated as “unclear.” For the completeness of outcome data, selective reporting of results, and the presence of other sources of bias, all studies were rated as “low risk.” Overall, the primary source of bias in the included studies stemmed from inadequate reporting of key details in the experimental design and implementation process, which may limit the strength of the conclusions drawn from the combined results. Detailed risk of bias assessment results are presented in Figure 2.

### 3.4 Meta-analysis results

### 3.4.1 Behavioral and cognitive outcomes

**FIGURE 3:**
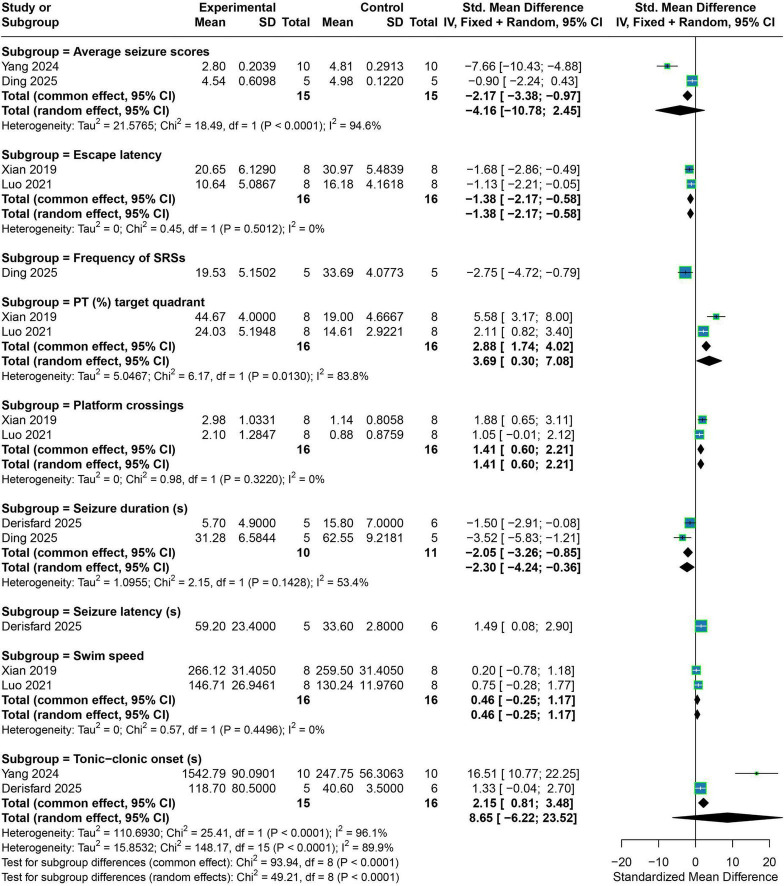
Forest plots of the meta-analyses for behavioral and cognitive outcomes. The figure shows the effects of exosome treatment on behavioral and cognitive outcomes in animal models of epilepsy, including Average seizure scores, Escape latency, Frequency of SRSs, PT (%) target quadrant, Platform crossings, Seizure duration (s), Seizure latency (s), Swim speed, and Tonic–clonic onset (s). Square size reflects the weight of each study in the pooled effect; horizontal lines represent 95% confidence intervals; diamonds represent pooled effects. Negative values indicate that exosome treatment reduced the outcome, and positive values indicate an increase.

Exosome treatment demonstrated significant improvements across multiple key behavioral and cognitive endpoints. Specifically, exosome administration significantly reduced escape latency in the Morris water maze (SMD = −1.38, 95% CI −2.17 to −0.58; fixed-effects model), decreased the frequency of spontaneous recurrent seizures (SMD = −1.38, 95% CI −2.17 to −0.58; single study), and shortened seizure duration (SMD = −2.30, 95% CI −4.24 to −0.36; random-effects model). In addition, treatment significantly increased the percentage of time spent in the target quadrant (SMD = 3.69, 95% CI 0.30–7.08; random-effects model), enhanced the number of platform crossings (SMD = 1.41, 95% CI 0.60–2.21; fixed-effects model), and prolonged seizure latency (SMD = 1.49, 95% CI 0.08–2.90; single study). In contrast, no statistically significant between-group differences were observed in tonic–clonic seizure onset latency (SMD = 8.65, 95% CI −6.22 to 23.52; random-effects model), average seizure scores (SMD = −4.16, 95% CI −10.78 to 2.45; random-effects model), or swimming speed (SMD = 0.46, 95% CI −0.25 to 1.17; fixed-effects model) (Figure 3).

#### 3.4.2 Neuronal and glial cell–related outcomes

**FIGURE 4:**
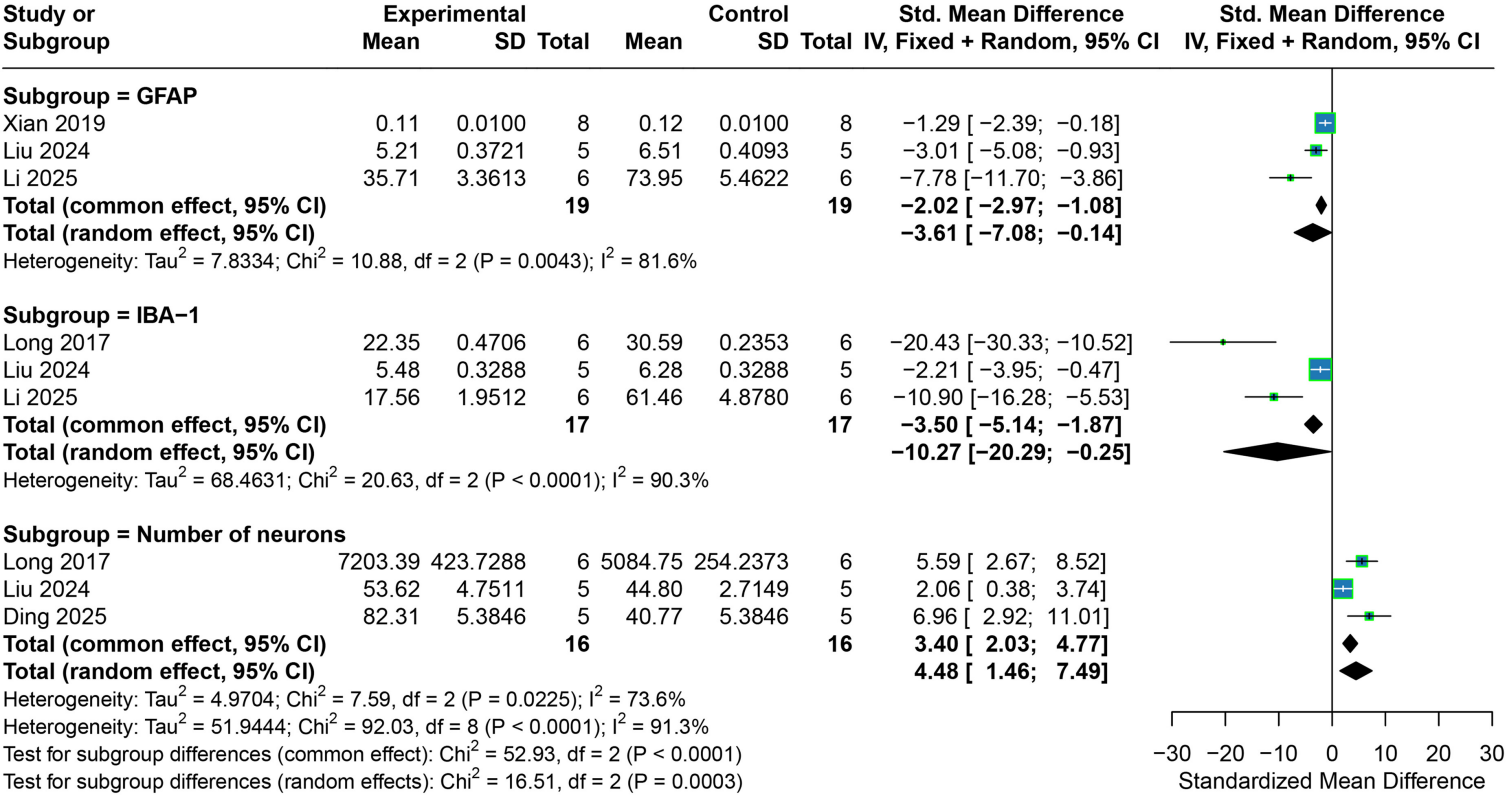
Forest plots of the meta-analyses for neuron- and glia-related outcomes. The figure shows the effects of exosome treatment on GFAP expression, IBA-1 expression, and hippocampal neuron number. Square size reflects the weight of each study in the pooled effect; horizontal lines represent 95% confidence intervals; diamonds represent pooled effects. Negative values indicate decreases, and positive values indicate increases.

Exosome therapy exerted clear anti-inflammatory and neuroprotective effects in preclinical epilepsy models. For GFAP, three studies exhibited substantial heterogeneity (*I*^2^ = 81.6%). A random-effects model demonstrated a significant reduction in GFAP expression following exosome treatment (SMD = −3.61, 95% CI −7.08 to −0.14), indicating suppression of astrocytic activation. Similarly, analysis of the microglial marker IBA-1 across three studies revealed very high heterogeneity (*I*^2^ = 90.3%), yet the random-effects model showed a significant decrease in IBA-1 expression in the treatment group (SMD = −10.27, 95% CI −20.29 to −0.25). For neuronal number, three studies demonstrated moderate-to-high heterogeneity (I^2^ = 73.6%). Pooled analysis using a random-effects model indicated that exosome therapy significantly increased neuronal counts (SMD = 4.48, 95% CI 1.46–7.49) (Figure 4).

#### 3.4.3 TNF-α levels

**FIGURE 5:**
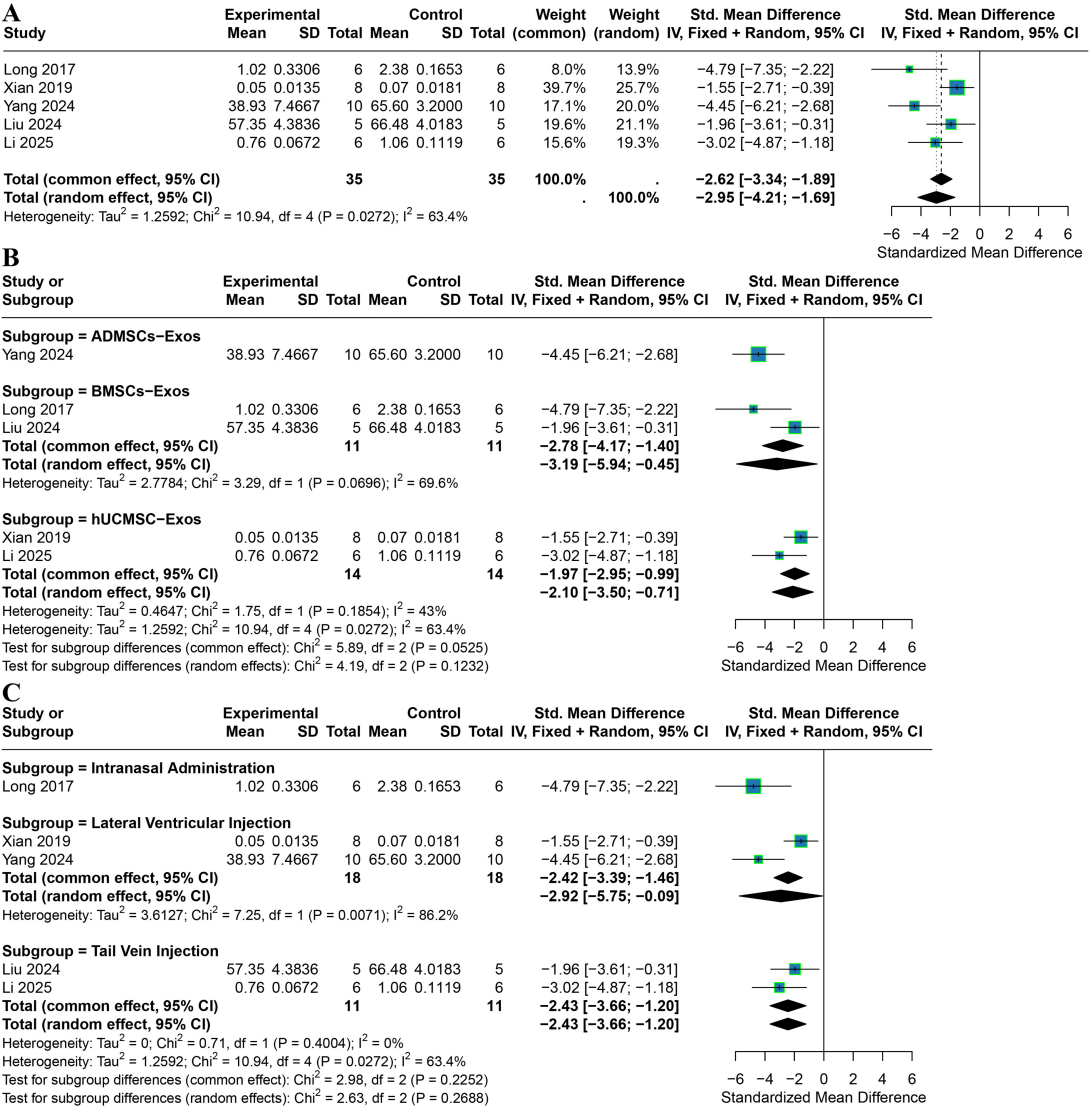
Meta-analysis results for TNF-α. **(A)** The overall forest plot shows that exosome treatment significantly reduced TNF-α levels (five studies). **(B)** Subgroup forest plot stratified by exosome source (ADMSCs-Exos, BMSCs-Exos, hUCMSCs-Exos). **(C)** Subgroup forest plot stratified by administration route (intracerebroventricular injection, intranasal administration, tail vein injection). Square size reflects the weight of each study in the pooled effect; horizontal lines represent 95% confidence intervals; diamonds represent pooled effects. *P*-values for tests of subgroup differences are shown beneath each subgroup plot.

Five studies were included in the meta-analysis of TNF-α expression. The pooled analysis showed moderate heterogeneity (*I*^2^ = 63.4%). A random-effects model revealed that exosome treatment significantly reduced TNF-α levels (SMD = −2.95, 95% CI −4.21 to −1.69) (Figure 5A).

Subgroup analysis by exosome source showed that the ADMSC-Exos subgroup (1 study) demonstrated a pronounced reduction in TNF-α (SMD = −4.45, 95% CI −6.21 to −2.68). The BMSC-Exos subgroup (2 studies) exhibited moderate heterogeneity (*I*^2^ = 69.6%), with a significant pooled effect under a random-effects model (SMD = −3.19, 95% CI −5.94 to −0.45). The hUCMSC-Exos subgroup (2 studies) showed low heterogeneity (*I*^2^ = 43%), and a fixed-effects model confirmed a significant reduction in TNF-α (SMD = −1.97, 95% CI −2.95 to −0.99). No statistically significant differences were detected between subgroups (*P* > 0.05) (Figure 5B).

Subgroup analysis by administration route indicated high heterogeneity for intracerebroventricular injection (2 studies; *I*^2^ = 86.2%), with a significant reduction in TNF-α under a random-effects model (SMD = −2.92, 95% CI −5.75 to −0.09). Intranasal administration (1 study) showed a strong effect (SMD = −4.79, 95% CI −7.35 to −2.22). Tail vein injection (2 studies) showed no heterogeneity (*I*^2^ = 0%), and a fixed-effects model demonstrated a significant reduction (SMD = −2.43, 95% CI −3.66 to −1.20). Between-subgroup differences were not statistically significant (*P* > 0.05) (Figure 5C).

#### 3.4.4 IL-1β levels

**FIGURE 6:**
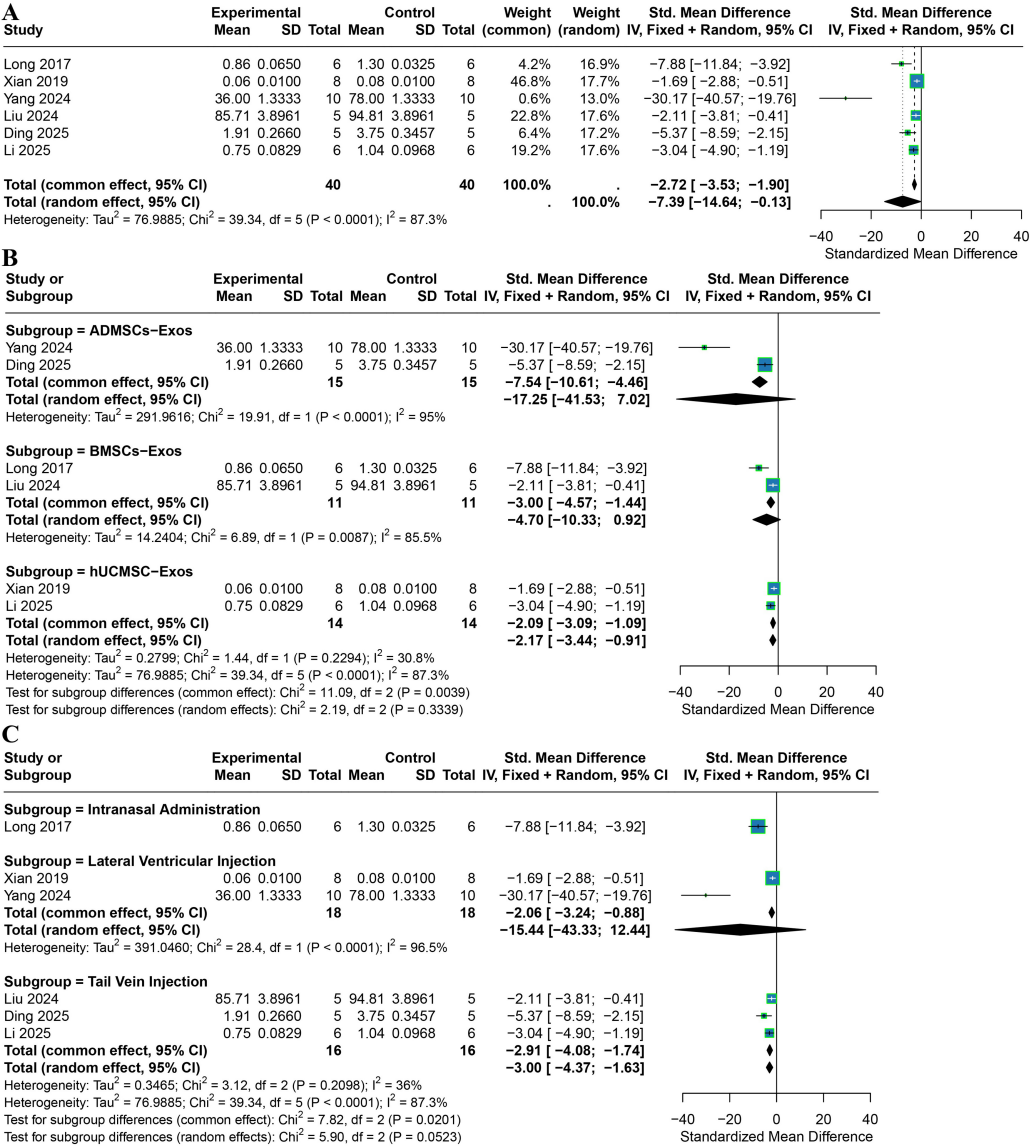
Meta-analysis results for IL-1β. **(A)** The overall-effect forest plot shows that exosome treatment significantly reduced IL-1β levels (six studies). **(B)** Subgroup forest plot stratified by exosome source (ADMSCs-Exos, BMSCs-Exos, hUCMSCs-Exos). **(C)** Subgroup forest plot stratified by administration route (intracerebroventricular injection, tail vein injection, intranasal administration). Square size reflects the weight of each study in the pooled effect; horizontal lines represent 95% confidence intervals; diamonds pooled effects. *P*-values for tests of subgroup differences are shown beneath each subgroup plot.

Six studies were included in the overall meta-analysis of IL-1β levels. Given the high heterogeneity (*I*^2^ = 87.3%), a random-effects model was applied. Exosome treatment significantly reduced IL-1β expression (SMD = −7.39, 95% CI −14.64 to −0.13) (Figure 6A).

Subgroup analysis by exosome source revealed extremely high heterogeneity in the ADMSC-Exos subgroup (2 studies; I^2^ = 95.0%), with no statistically significant effect (random-effects SMD = −17.25, 95% CI −41.53 to 7.02). Similarly, the BMSC-Exos subgroup (2 studies) showed high heterogeneity (*I*^2^ = 85.5%) and no significant pooled effect (random-effects SMD = −4.70, 95% CI −10.33 to 0.92). In contrast, the hUCMSC-Exos subgroup (2 studies) demonstrated low heterogeneity (*I*^2^ = 30.8%), and a fixed-effects model indicated a significant reduction in IL-1β (SMD = −2.17, 95% CI −3.44 to −0.91). Between-subgroup differences were significant under the fixed-effects model (*P* = 0.0039) but not under the random-effects model (*P* = 0.3339) (Figure 6B).

Subgroup analysis by administration route showed extremely high heterogeneity for intracerebroventricular injection (2 studies; *I*^2^ = 96.5%), with no significant effect (random-effects SMD = −15.44, 95% CI −43.33 to 12.44). Tail vein injection (3 studies) showed lower heterogeneity (*I*^2^ = 36.0%), and a fixed-effects model demonstrated a significant reduction in IL-1β (SMD = −3.00, 95% CI −4.37 to −1.63). Intranasal administration (1 study) also showed a marked reduction (SMD = −7.88, 95% CI −11.84 to −3.92). Between-subgroup differences were significant under the fixed-effects model (*P* = 0.0201) and marginally significant under the random-effects model (*P* = 0.0523) (Figure 6C).

#### 3.4.5 Publication bias

**FIGURE 7:**
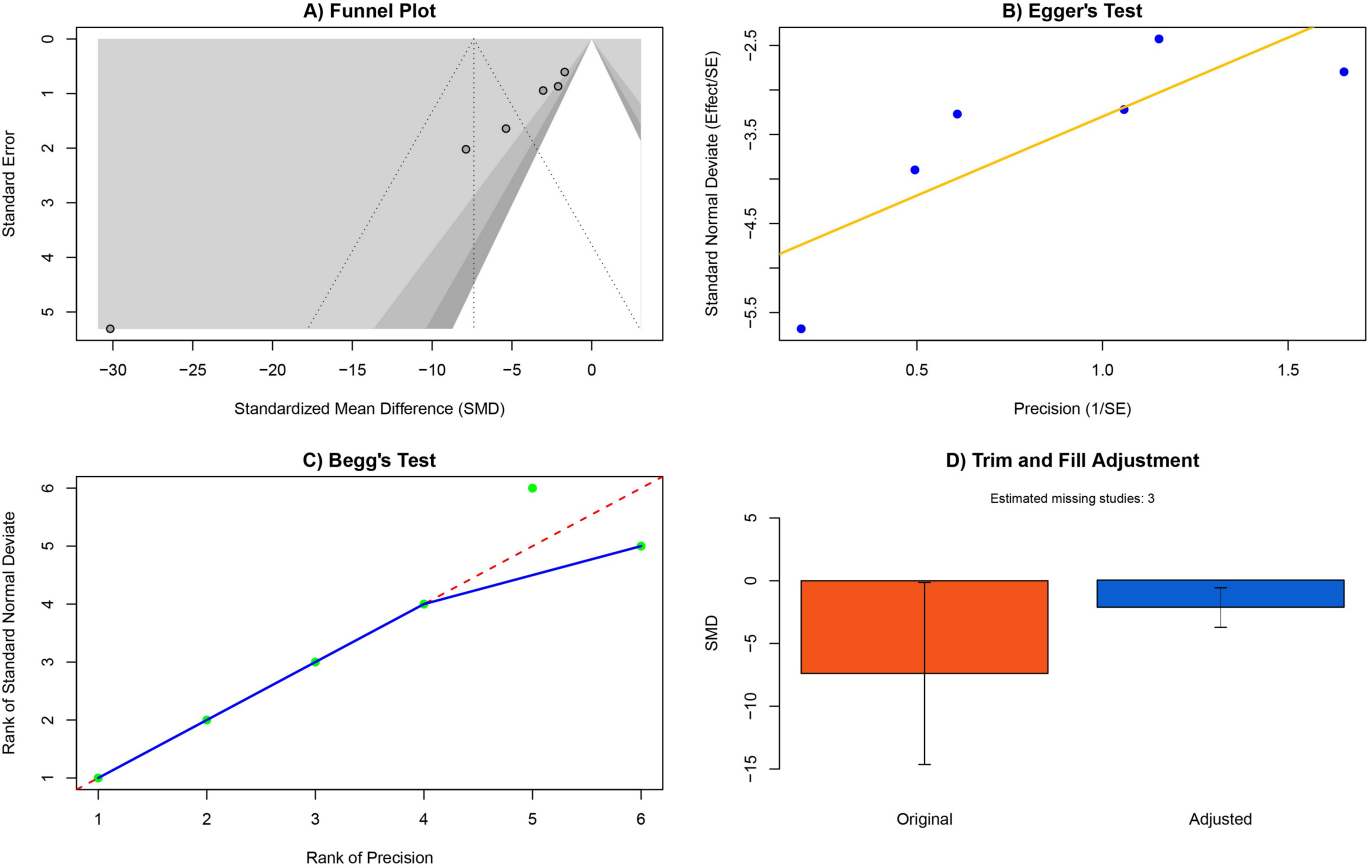
Assessment of publication bias. **(A)** The funnel plot shows the assessment of publication bias for the IL-1β outcome, with an asymmetric distribution. **(B)** Results of Egger’s linear regression test. **(C)** Results of Begg’s rank correlation test. **(D)** Trim-and-fill adjustment, indicating that three missing studies would be required to correct for publication bias.

Publication bias for the IL-1β outcome was systematically assessed using multiple approaches. Visual inspection of the funnel plot suggested potential asymmetry. Quantitative analyses further confirmed significant publication bias, as indicated by both Egger’s linear regression test (*P* < 0.05) and Begg’s rank correlation test (*P* < 0.05). To account for this bias, the trim-and-fill method was applied, estimating the presence of three potentially missing studies. Before adjustment, the pooled effect size was SMD = −7.39 (95% CI −14.64 to −0.13). After trim-and-fill correction, the effect size attenuated to SMD = −2.08 (95% CI −3.70 to −0.56). Importantly, the lower bound of the 95% confidence interval remained below zero, indicating that the effect of exosome therapy in reducing IL-1β levels remained statistically significant despite bias correction. These findings suggest that the conclusions of this meta-analysis are reasonably robust, although the magnitude of the unadjusted effect should be interpreted with caution (Figure 7).

## 4 Discussion

This study represents the first systematic review and meta-analysis to quantitatively synthesize the efficacy of exosome therapy for epilepsy in preclinical animal experiments. The comprehensive analysis indicates that exosome interventions have clear and broad therapeutic effects in improving seizure behaviors, protecting cognitive function, alleviating neuroinflammation, and promoting neuronal survival. However, the significant heterogeneity observed among studies suggests that the therapeutic effects of exosomes are modulated by various factors.

### 4.1 Comprehensive assessment of main efficacy

Our meta-analysis confirmed the considerable potential of exosomes as a multitarget therapeutic strategy. At the behavioral level, exosome treatment significantly shortened seizure latency and seizure duration and reduced the frequency of spontaneous recurrent seizures. Taken together, these findings suggest that exosomes not only raise the threshold for acute seizures but may also exert disease-modifying effects by delaying or inhibiting epileptogenesis (Arvin et al., 2025; Derisfard et al., 2025; Ding et al., 2025; Long et al., 2017). The improvement in cognitive function is particularly noteworthy, as evidenced by the reduced escape latency and increased time spent in the target quadrant during the Morris water maze, clearly indicating that exosomes effectively mitigate hippocampal-dependent learning and memory impairments associated with epilepsy (Ledri and Kokaia, 2026). Importantly, the lack of significant differences in swimming speed across groups rules out potential confounding effects of motor dysfunction on cognitive test outcomes, further reinforcing the conclusion of exosomes’ direct protective effects on cognitive function.

Neuropathological indicators provide cellular and molecular explanations for the aforementioned behavioral improvements. The significant increase in neuronal counts in the hippocampus following exosome treatment directly confirms its robust neuroprotective capabilities. Concurrently, the marked decrease in the expression of the astrocytic marker GFAP and the microglial marker IBA-1, along with reductions in key pro-inflammatory cytokines TNF-α and IL-1β, collectively demonstrate the effectiveness of exosomes in mediating therapeutic effects through the inhibition of neuroinflammatory cascades (Xian et al., 2019). Neuroinflammation is a core driver of epilepsy onset and progression, exacerbating the disease process through mechanisms such as disrupting neuronal excitability, compromising blood-brain barrier integrity, and inducing oxidative stress (Hollis and Lukens, 2025; Ravizza et al., 2024). The diverse bioactive molecules carried by exosomes may synergistically regulate the abnormal activation of microglia and astrocytes while inhibiting key pro-inflammatory signaling pathways, including NOD-, LRR- and pyrin domain-containing protein 3 (NLRP3) inflammasome, nuclear factor kappa B (NF-κB), and high mobility group box 1 (HMGB1)/Toll-like receptor 4 (TLR4), thereby alleviating inflammation-induced neuronal damage (Liu et al., 2024; Zhang et al., 2022).

However, a complete understanding of efficacy must also include a careful appraisal of negative findings. In this study, the effects of exosome treatment on delaying tonic–clonic onset and improving average seizure scores did not reach statistical significance (Arvin et al., 2025). This suggests that the anticonvulsant effects of exosomes may be contingent on boundary conditions or exhibit target selectivity. For example, exosomes may significantly influence certain aspects of seizure severity (e.g., duration) but have limited impact on the onset of specific high-intensity components (e.g., the tonic–clonic phase), which may relate to the timing window of intervention, pathway specificity, or differences in the neural circuits underlying distinct seizure types (Arvin et al., 2025). As a composite measure, the average seizure score may also lack sufficient sensitivity to capture exosome-related improvements in specific behavioral components. In addition, exosome-mediated modulation of seizures may involve dual mechanisms: on the one hand, exosomes may reduce acute seizure susceptibility by suppressing neuroinflammation and oxidative stress, thereby raising the neuronal excitability threshold (Liu et al., 2024; Xian et al., 2019); on the other hand, in the chronic phase, exosomes may disrupt the formation and consolidation of epileptic networks promoting neuroregeneration and synaptic remodeling and by inhibiting molecular pathways implicated in epileptogenesis (e.g., mTOR, Mammalian Target of Rapamycin) (Łukasiuk and Lasoń, 2023; Luo et al., 2021; Zhao et al., 2023). These two mechanisms are not mutually exclusive and may act synergistically at different stages. Together, these findings underscore the need for future studies to more finely delineate the specific effects of exosomes across different dimensions of seizures and to distinguish their differential roles in acute anticonvulsant activity versus chronic anti-epileptogenic effects.

### 4.2 Analysis of sources of heterogeneity and key influencing factors

Despite the overall significant efficacy, several outcome measures (such as GFAP, IBA-1, and IL-1β) exhibited moderate to high heterogeneity (*I*^2^ > 50%). This does not imply that the results are unreliable; rather, it reflects the diversity in design parameters across current preclinical studies, which is one of the core issues this meta-analysis aims to elucidate.

First, the cellular source of exosomes is a key determinant of differences in effect size. Among the eight included studies, six (75%) used exosomes derived from human MSCs—three from umbilical cord, two from adipose tissue, and one from bone marrow—whereas the remaining two (25%) used exosomes derived from mouse MSCs/ADSCs. Our subgroup analyses showed that exosomes from different sources all tended to reduce inflammatory mediators, but the magnitude of effect and the degree of within-subgroup consistency differed. For instance, in the IL-1β analysis, the hUCMSCs-Exos subgroup showed lower heterogeneity and a consistent significant effect, whereas the ADMSCs-Exos subgroup exhibited extremely high heterogeneity. Notably, human- versus mouse-derived exosomes may differ fundamentally in immunogenicity, efficiency of crossing the blood–brain barrier, and interactions with the host neural microenvironment, yet no direct head-to-head comparisons are currently available. This may reflect inherent differences in the miRNA and protein cargo profiles of exosomes released by MSCs from different tissues, leading to regulation of specific inflammatory pathways varying strength or specificity (Bi et al., 2022; Wang et al., 2022). For example, hUCMSCs-Exos are enriched in antioxidant miRNAs (e.g., miR-215-5p, miR-424-5p) and act by activating the Nrf2 (Nuclear Factor Erythroid 2-Related Factor 2) antioxidant defense system (Luo et al., 2021), whereas Liu et al. reported that engineered exosomes overexpressing miR-129-5p specifically target the HMGB1/TLR4 axis (Liu et al., 2024). Therefore, future studies should not simply treat all MSC-derived exosomes as equivalent, but should further clarify the links between cargo composition and functional characteristics.

Second, the route of administration significantly influences therapeutic efficacy. Subgroup analysis indicated that tail vein injection and intranasal administration resulted in much lower within-group heterogeneity when reducing TNF-α and IL-1β compared to the overall analysis or the lateral ventricle injection route. This suggests that these two systemic/non-invasive administration methods may offer more consistent biodistribution and pharmacokinetic profiles. Exosomes administered via tail vein injection must traverse the systemic circulation and cross the blood-brain barrier, while intranasal administration may allow for direct or rapid entry into the brain via the olfactory or trigeminal nerves, both avoiding the trauma and local microenvironment disturbances associated with craniotomy (Mehdizadeh et al., 2025; Zheng et al., 2024). In contrast, while lateral ventricle injection ensures high concentrations of exosomes in cerebrospinal fluid, the procedure itself may introduce additional inflammatory variables, and the uneven distribution of exosomes within brain tissue could account for the substantial variability in results within that subgroup.

It should be noted that, limited by the number of included studies, we conducted subgroup analyses by exosome source and administration route only for inflammatory outcomes with relatively sufficient study numbers (TNF-α and IL-1β). For behavioral and cognitive outcomes (e.g., escape latency, platform crossings, seizure duration), most measures were reported by only one to two studies, and the sample size was insufficient to support meaningful stratification and statistical inference by source or route. For example, escape latency and platform crossings were each reported in only two studies; further stratification by source or route would leave only one study per subgroup, precluding calculation of pooled effect estimates and confidence intervals. Therefore, systematic subgroup analyses by source and route not be performed for all outcomes.

It is important to note that, due to the limited number of included studies, this research could not perform a two-factor interaction subgroup analysis of exosome source and administration route. For example, we could not compare the advantages of different administration routes while controlling for exosome type (e.g., all being hUCMSC-Exos); conversely, the same limitation applies in reverse. This constraint makes it challenging to fully disentangle the independent contributions of these two critical variables. However, when grouping based solely on source or route, the significant reduction in heterogeneity within some subgroups strongly suggests that both are major contributors to overall heterogeneity and must be considered as core parameters when optimizing treatment strategies. Additionally, the vast differences in exosome dosage (ranging from 15 to 350 μg) represent another key variable that could not be quantitatively merged or directly compared in this meta-analysis. The absence of a clear dose-response relationship remains a knowledge gap in the current field. Insufficient doses may fail to elicit therapeutic effects, while the potential for “spillover effects” or adverse reactions at excessively high doses remains unknown.

### 4.3 Cautious evaluation of evidence strength and bias

In interpreting the positive conclusions drawn above, it is essential to carefully consider the methodological foundations supporting these findings. The risk of bias assessment for the included studies revealed that the majority exhibited insufficient reporting on critical methodological aspects such as random sequence generation, allocation concealment, and implementation blinding, resulting in a classification of “unclear risk.” This widespread methodological reporting deficiency may systematically overestimate the magnitude of treatment effects. Furthermore, the current studies may be subject to publication bias and small sample effects. Although the effect remained significant after correction via the trim-and-fill method, confirming the robustness of the core conclusions, this also raises concerns about the potential for “positive result bias” in published studies, suggesting that the true effect size may be more conservative. These findings underscore the importance of adhering to and thoroughly reporting methodological standards, as recommended by tools like SYRCLE, in preclinical research to enhance the overall quality and reliability of future evidence.

### 4.4 Limitations of the study

This study has the following limitations: (1) The number of included animal experiments remains small (only eight), limiting the depth and statistical power of subgroup analyses; in particular, we could not perform subgroup analyses by exosome source and administration route for behavioral and cognitive outcomes, nor could we conduct multifactor interaction analyses. (2) Methodological reporting in the included studies was generally inadequate; notably, randomization, allocation concealment, and blinding were frequently rated as “unclear risk,” which may have led to overestimation of treatment effects. (3) Of the eight studies, only one directly assessed chronic spontaneous recurrent seizures using video-EEG monitoring; most focused on acute-phase outcomes or chronic pathological/cognitive outcomes, resulting in limited strength of evidence for the anti-epileptogenesis effects of exosomes. (4) The relative concentration of animal models (mainly chemically induced), strains (predominantly C57BL/6), and sex (predominantly male) restricts extrapolation to broader contexts (e.g., genetic epilepsy, female animals). (5) Characterization of exosomes (e.g., size, markers, cargo) reported with varying levels of detail across original studies, and species differences—two studies used mouse-derived exosomes and six used human-derived exosomes—may have introduced additional heterogeneity. (6) This meta-analysis primarily compared exosomes with control conditions and did not include AEDs as positive comparators for indirect comparison; therefore, we could not directly evaluate the advantages of exosomes relative to current standard treatments. (7) Clinical translation of exosome therapy still faces substantial practical challenges, including difficulties in standardizing preparation, markedly higher production costs than conventional AEDs, complex quality control for large-scale manufacturing, and limited accessibility in low- and middle-income countries (Low- and Middle-Income Countries, LMICs) (Bi et al., 2022; Wang et al., 2022). These issues have not been adequately evaluated in current preclinical studies, yet they are key determinants of whether exosome therapy can ultimately benefit people with epilepsy worldwide.

In light of these limitations, future research should be optimized in several respects. First, in selecting animal models, chronic SRS models (e.g., chronic epilepsy after pilocarpine- or kainic acid–induced status epilepticus) should be prioritized, with long-term (≥ 2 weeks) video-EE monitoring to more faithfully recapitulate the pathological course of clinically refractory epilepsy and to rigorously evaluate the anti-epileptogenic effects of exosomes. Second, in study design, exosome preparation protocols should be standardized as much as possible (e.g., unified culture conditions for the source cells, isolation methods, and characterization criteria), with clear differentiation and reporting of human- versus mouse-derived exosomes, and multiple dose groups should be included (drawing on the dose range in this meta-analysis, a low dose such as 10–20 μg, a medium dose such as 50–100 μg, and a high dose such as 200–300 μg) to define the dose–response relationship. Third, AED positive-control groups (e.g., sodium valproate, levetiracetam) should be incorporated to directly compare exosomes with existing drugs in seizure control, cognitive protection, and suppression of neuroinflammation. Finally, with respect to administration route, non-invasive systemic approaches such as intranasal delivery or tail vein injection should be prioritized, and different routes should be compared within the same study to identify the optimal delivery strategy.

## 5 Conclusion

This study provides robust quantitative evidence for the preclinical efficacy of exosome therapy in epilepsy for the first time. Exosomes demonstrate significant comprehensive therapeutic benefits in improving seizure behaviors, protecting cognitive function, inhibiting neuroinflammation, and reducing neuronal loss. Although efficacy is significantly influenced by the source of exosomes, administration route, and dosage, leading to heterogeneity among studies—these factors are precisely the key aspects for optimizing future treatment strategies. In summary, this research lays a solid preclinical evidence foundation for the design of subsequent animal experiments and the advancement of exosome therapy toward clinical trials for epilepsy.

## Data Availability

The original contributions presented in the study are included in the article/Supplementary material, further inquiries can be directed to the corresponding author.
