# Supplementary material for: Exosome-based therapy for epilepsy: a systematic review and meta-analysis of preclinical studies
Source: Front Neurosci. 2026 May 15;20:1824183. doi: 10.3389/fnins.2026.1824183 (PMC13219301; doi:10.3389/fnins.2026.1824183)
Supplement: Supplementary file 1 [file Table_1.docx]

**Exosome-Based Therapy for Epilepsy: A Systematic Review and Meta-Analysis of Preclinical Studies**

**Supplementary Table 1. Search strategy**

| **1.PubMed**  #1: "exosome*"[Title/Abstract] OR "exosomes"[Title/Abstract] OR "exosomal"[Title/Abstract] OR "extracellular vesicle"[Title/Abstract] OR "extracellular vesicles"[Title/Abstract] OR "extracellular particle"[Title/Abstract] OR "extracellular particles"[Title/Abstract] OR "microvesicle"[Title/Abstract] OR "microvesicles"[Title/Abstract] OR "Shedding Microvesicle"[Title/Abstract] OR "Shedding Microvesicles"[Title/Abstract] OR "Secretory Vesicle"[Title/Abstract] OR "Secretory Vesicles"[Title/Abstract] OR "Cell-Derived Microparticle"[Title/Abstract] OR "Cell-Derived Microparticles"[Title/Abstract] OR "microbubble"[Title/Abstract] OR "microbubbles"[Title/Abstract] OR "apoptotic body"[Title/Abstract] OR "apoptosis bodies"[Title/Abstract]  #2: "Exosomes"[MeSH Terms] OR "Extracellular Vesicles"[MeSH Terms] OR "Cell-Derived Microparticles"[MeSH Terms] OR "Microbubbles"[MeSH Terms]  #3: #1 OR #2  #4: "Epilepsy"[Title/Abstract] OR "Seizure"[Title/Abstract] OR "Seizures"[Title/Abstract] OR "Epileptic seizure"[Title/Abstract] OR "Epileptic seizures"[Title/Abstract] OR "Seizure disorder"[Title/Abstract] OR "Refractory epilepsy"[Title/Abstract] OR "Drug resistant epilepsy"[Title/Abstract] OR "Temporal lobe epilepsy"[Title/Abstract] OR "Status epilepticus"[Title/Abstract] OR "Epileptogenesis"[Title/Abstract]  #5: "Epilepsy"[MeSH Terms] OR "Seizures"[MeSH Terms]  #6: #4 OR #5  #7: #3 AND #6  **2. WOS**  (TS=("exosome" OR "exosomes" OR "exosomal" OR "extracellular vesicle" OR "extracellular vesicles" OR "extracellular particle" OR "extracellular particles" OR "microvesicle" OR "microvesicles" OR "Shedding Microvesicle" OR "Shedding Microvesicles" OR "Secretory Vesicle" OR "Secretory Vesicles" OR "Cell-Derived Microparticle" OR "Cell-Derived Microparticles" OR "microbubble" OR "microbubbles" OR "apoptotic body" OR "apoptosis bodies")) AND TS=(Epilepsy OR Seizure OR Seizures OR "Epileptic seizure" OR "Epileptic seizures" OR "Seizure disorder" OR "Refractory epilepsy" OR "Drug resistant epilepsy" OR "Temporal lobe epilepsy" OR "Status epilepticus" OR Epileptogenesis) AND (Refinement basis:Literature Type: Thesis or Review Paper or Other or Online Publication or Clinical Trial)  **3. Embase**  #1: 'exosome*':ti,ab,kw OR 'exosomes':ti,ab,kw OR 'exosomal':ti,ab,kw OR 'extracellular vesicle':ti,ab,kw OR 'extracellular vesicles':ti,ab,kw OR 'extracellular particle':ti,ab,kw OR 'extracellular particles':ti,ab,kw OR 'microvesicle':ti,ab,kw OR 'microvesicles':ti,ab,kw OR 'shedding microvesicle':ti,ab,kw OR 'shedding microvesicles':ti,ab,kw OR 'secretory vesicle':ti,ab,kw OR 'secretory vesicles':ti,ab,kw OR 'cell-derived microparticle':ti,ab,kw OR 'cell-derived microparticles':ti,ab,kw OR 'microbubble':ti,ab,kw OR 'microbubbles':ti,ab,kw OR 'apoptotic body':ti,ab,kw OR 'apoptosis bodies':ti,ab,kw  #2: 'exosome'/exp  #3: 'exosomes'/exp  #4: 'extracellular vesicle'/exp  #5: 'microvesicle'/exp  #6: 'secretory vesicle'/exp  #7: 'cell-derived microparticle'/exp  #8: 'microbubble'/exp  #9: 'apoptotic body'/exp  #10: #1 OR #2 OR #3 OR #4 OR #5 OR #6 OR #7 OR #8 OR #9  #11: epilepsy:ti,ab,kw OR seizure:ti,ab,kw OR seizures:ti,ab,kw OR 'epileptic seizure':ti,ab,kw OR 'epileptic seizures':ti,ab,kw OR 'seizure disorder':ti,ab,kw OR 'refractory epilepsy':ti,ab,kw OR 'drug resistant epilepsy':ti,ab,kw OR 'temporal lobe epilepsy':ti,ab,kw OR 'status epilepticus':ti,ab,kw OR epileptogenesis:ti,ab,kw  #12: 'epilepsy'/exp  #13: 'seizure'/exp  #14: 'epileptic seizure'/exp  #15: 'seizure disorder'/exp  #16: 'refractory epilepsy'/exp  #17: 'drug resistant epilepsy'/exp  #18: 'temporal lobe epilepsy'/exp  #19: 'status epilepticus'/exp  #20: 'epileptogenesis'/exp  #21: #11 OR #12 OR #13 OR #14 OR #15 OR #16 OR #17 OR #18 OR #19 OR #20  #22: #10 AND #21  #23: #22 AND ('article'/it OR 'article in press'/it OR 'clinical trial'/it OR 'preprint'/it OR 'review'/it)  **4. Scopus**  TITLE-ABS-KEY ( "exosome" OR "exosomes" OR "exosomal" OR "extracellular vesicle" OR "extracellular vesicles" OR "extracellular particle" OR "extracellular particles" OR "microvesicle" OR "microvesicles" OR "Shedding Microvesicle" OR "Shedding Microvesicles" OR "Secretory Vesicle" OR "Secretory Vesicles" OR "Cell-Derived Microparticle" OR "Cell-Derived Microparticles" OR "microbubble" OR "microbubbles" OR "apoptotic body" OR "apoptosis bodies" ) AND TITLE-ABS-KEY ( Epilepsy OR Seizure OR Seizures OR "Epileptic seizure" OR "Epileptic seizures" OR "Seizure disorder" OR "Refractory epilepsy" OR "Drug resistant epilepsy" OR "Temporal lobe epilepsy" OR "Status epilepticus" OR Epileptogenesis ) AND ( LIMIT-TO ( DOCTYPE , "ar" ) OR LIMIT-TO ( DOCTYPE , "re" ) )  **5. CNKI**  Exosome OR Extracellular Vesicle OR Exosome-like Vesicle OR Microvesicle Epilepsy OR Seizures OR Convulsions OR Tonic-Clonic Seizures OR Grand Mal Epilepsy SU %= (‘Exosome’) OR (‘Extracellular Vesicle’) OR (‘Exosome-like Vesicle’) OR (‘Microvesicle’) AND SU %= (‘Epilepsy’) OR (‘Seizures’) OR (‘Convulsions’) OR (‘Tonic-Clonic Seizures’) OR (‘Grand Mal Epilepsy’)  **6. Wanfang**  Topic: (Exosome OR Extracellular Vesicle OR Exosome-like Vesicle OR Microvesicle) AND Topic: (Epilepsy OR Seizures OR Convulsions OR Tonic-Clonic Seizures OR Grand Mal Epilepsy)  **7. VIP**  (SU=Exosome OR SU=Extracellular Vesicle OR SU=Exosome-like Vesicle OR SU=Microvesicle) AND (SU=Epilepsy OR SU=Seizures OR SU=Convulsions OR SU=Tonic-Clonic Seizures OR SU=Grand Mal Epilepsy)  **8. SinoMed**  #1: (“Exosome”[Unweighted: Extended]) OR “Extracellular Vesicle”[Unweighted: Extended] #2: “Exosome”[Common Fields: Intelligent] OR “Extracellular Vesicle”[Common Fields: Intelligent] OR “Exosome-like Vesicle”[Common Fields: Intelligent] OR “Microvesicle”[Common Fields: Intelligent] #3: #1 OR #2 #4: “Epilepsy”[Unweighted: Extended] #5: “Epilepsy”[Common Fields: Intelligent] OR “Seizures”[Common Fields: Intelligent] OR “Convulsions”[Common Fields: Intelligent] OR “Tonic-Clonic Seizures”[Common Fields: Intelligent] OR “Grand Mal Epilepsy”[Common Fields: Intelligent] #6: #4 OR #5 #7: #3 AND #6 |
| --- |

**Supplementary Table 2. Basic characteristics of the included studies**

| No. | Author | Year | Country | Research Type | Animal Characteristics | | | | Sample Size | Modeling Method | Stem Cells | | Transplantation Route | Transplantation Dose | Control Group |
| --- | --- | --- | --- | --- | --- | --- | --- | --- | --- | --- | --- | --- | --- | --- | --- |
|  |  |  |  |  | Species | Sex | Weight | Age | Experimental Group/Control Group |  | Type | Source |  |  |  |
| 1 | Liu | 2024 | China | RCT | C57BL/6 J mice | Male | 20–25 g | 8 weeks | 5/5 | Intraperitoneal injection of kainic acid to induce SE | BMSCs-Exos | Extracted from mouse bone marrow | Tail vein injection | 100 μg | PBS |
| 2 | Derisfard | 2025 | Iran | RCT | Mice | Male | 22–28 g | 8–12 weeks | 5/6 | Intraperitoneal injection of pentylene tetrazole to induce acute seizures | ADMSCs-Exos | Human adipose tissue | Tail vein injection | 15 µg | PBS |
| 3 | Ding | 2025 | China | RCT | C57BL/6 mice | Male | 20–30 g | 6–8 weeks | 5/5 | Hippocampal injection of kainic acid to induce temporal lobe epilepsy model | ADMSCs-Exos | Human adipose tissue | Unilateral cortical injection | 300 μg | Saline solution |
| 4 | Long | 2017 | United States | RCT | Mice | / | / | / | 6/6 | Pilocarpine-induced SE | BMSCs-Exos | Human bone marrow-derived mesenchymal stem cells | Tail vein injection | 30 μg | PBS |
| 5 | Che | 2025 | China | RCT | C57BL/6 mice | Male | 18–22 g | 6–8 weeks | 36/12 | Pilocarpine-induced SE | hUCMSC-Exos | Human umbilical cord mesenchymal stem cells | Tail vein injection | 350 μg | PBS |
| 6 | Yang | 2024 | China | RCT | C57BL/6 mice | Male | / | 8–10 weeks | 10/10 | Intraperitoneal injection of kainic acid to induce SE | ADMSCs-Exos | Mouse adipose-derived stem cells | Tail vein injection | NA | PBS |
| 7 | Xian | 2019 | China | RCT | C57B/6 mice | Male | / | 6–8 weeks | 8/8 | Pilocarpine-induced SE | hUCMSC-Exos | Human umbilical cord Wharton’s jelly | Intraperitoneal injection | 30 μg | PBS |
| 8 | Luo | 2021 | China | RCT | C57BL/6 mice | Male | / | 8–10 weeks | 8/8 | Intraperitoneal injection of picrotoxin to induce SE | hUCMSC-Exos | Human umbilical cord Wharton’s jelly | Tail vein injection | 50 μg | PBS |
